# Positron Emission Tomography to Improve Assessment of Interstitial Lung Disease in Patients With Systemic Sclerosis Eligible for Autologous Stem Cell Transplantation

**DOI:** 10.3389/fimmu.2022.923869

**Published:** 2022-07-05

**Authors:** Bo Broens, Conny J. van der Laken, Gerben J.C. Zwezerijnen, Esther J. Nossent, Lilian J. Meijboom, Julia Spierings, Jeska K. de Vries-Bouwstra, Jacob M. van Laar, Alexandre E. Voskuyl

**Affiliations:** ^1^ Department of Rheumatology and Clinical Immunology, Amsterdam UMC, Amsterdam, Netherlands; ^2^ Department of Radiology and Nuclear Medicine, Amsterdam UMC, Amsterdam, Netherlands; ^3^ Department of Pulmonary Medicine, Amsterdam UMC, Amsterdam, Netherlands; ^4^ Amsterdam Cardiovascular Sciences Research Institute, Amsterdam, Netherlands; ^5^ Department of Rheumatology and Clinical Immunology, University Medical Centre Utrecht, Utrecht, Netherlands; ^6^ Department of Rheumatology, Leiden University Medical Center, Leiden, Netherlands

**Keywords:** systemic sclerosis, scleroderma, interstitial lung disease, lung fibrosis, stem cell transplantation, positron emission tomography

## Abstract

Positron emission tomography (PET) is a promising technique to improve the assessment of systemic sclerosis associated interstitial lung disease (SSc-ILD). This technique could be of particular value in patients with severe diffuse cutaneous SSc (dcSSc) that are possibly eligible for autologous hematopoietic stem cell transplantation (aHSCT). aHSCT is a potentially effective therapy for patients with severe dcSSc and ILD, leading to stabilization or improvement of lung function. However, there is a high need to improve patient selection, which includes (1) the selection of patients with rapidly progressive ILD for early rather than last-resort aHSCT (2) the prediction of treatment response on ILD and (3) the understanding of the mechanism(s) of action of aHSCT in the lungs. As previous studies with ^18^F-FDG PET in SSc-ILD and other forms of ILD have demonstrated its potential value in predicting disease progression and reactivity to anti-inflammatory treatment, we discuss the potential benefit of using this technique in patients with early severe dcSSc and ILD in the context of aHSCT. In addition, we discuss the potential value of other PET tracers in the assessment of ILD and understanding the mechanisms of action of aHSCT in the lung. Finally, we provide several suggestions for future research.

## Introduction

Respiratory failure due to interstitial lung disease (ILD) is the primary cause of death in patients with systemic sclerosis (SSc) (1). Up to 80-90% of the patients with SSc develop some form of ILD and 10-year mortality in case of progression is estimated at 40% (2, 3). While some patients have no symptoms and remain stable over a long period of time, others can experience cough and breathlessness and deteriorate rapidly (3). Risk factors for rapid progressive ILD in SSc include diffuse cutaneous disease (dcSSc), anti-topo-isomerase I antibodies, shorter disease duration, older age at disease onset and male sex (3–5). In the past 10 years, several anti-inflammatory and anti-fibrotic treatments have been investigated and implemented in SSc-ILD. These treatments mainly stabilize or slow deterioration of ILD, while improvement of lung function and fibrotic ILD is hardly possible (6).

Autologous hematopoietic stem cell transplantation (aHSCT) is a potentially effective therapy for the treatment of severe dcSSc, with or without ILD. In the published randomized controlled trials (RCTs) for aHSCT in dcSSc to date, 79-97% patients had ILD. All three RCTs showed stabilization or improvement in lung volumes and extent of ILD, but less effect was seen on the diffusion capacity for carbon monoxide (DLCO) (7–9). Other, smaller and non-randomized studies, found similar results (10–13). However, as aHSCT carries a potential risk of treatment related mortality (TRM), ranging between 5-10% based on recent studies, it is often seen as a last resort treatment (11, 14). Therefore, it is essential to identify patients with rapidly progressive ILD who may qualify for early treatment, as TRM risk increases when patients lack sufficient reserve capacity. Also, it is important to select those patients that would benefit most from aHSCT, which is now difficult to predict. A small study specifically evaluated the change in extent of ILD after aHSCT and observed a more pronounced reduction of ILD extent in patients with more ground-glass opacities at baseline, which underlines that adequate timing of aHSCT is important to improve lung involvement (15).

Current pulmonary evaluation before aHSCT consists of pulmonary function tests, high resolution computed tomography (HRCT) and a right-heart catheterization. A right heart catheterization is performed to rule-out pulmonary hypertension, which is a common complication of SSc and has been associated with higher TRM in earlier trials (16). Pulmonary function tests, including forced vital capacity and DLCO, are most indicative for monitoring ILD. However, the use of DLCO is limited by its sensitivity to various other factors, including pulmonary infections, emphysema and congestive heart failure, which are common comorbidities in SSc-ILD (17). HRCT is a very sensitive tool to detect ILD, but depicts anatomical changes and cannot distinguish sufficiently between inflammatory and fibrotic activity or reversible and irreversible disease (18). For example, ground-glass opacities on HRCT might be a reflection of inflammation, interstitial edema and fibrotic tissue, and are thus not pathognomonic for inflammation.

As current tools to evaluate ILD have the preceding limitations, there is increasing interest to determine the use of molecular imaging with positron emission tomography (PET) in the management of patients with SSc-ILD. PET could guide in the aforementioned challenges due to its ability to non-invasively evaluate specific molecular processes in the lungs and by the availability of several tracers, different disease mechanisms can be investigated.

## Positron Emission Tomography

PET enables visualization of specific molecular processes or targets using (semi)selective radio ligands, i.e. PET tracers. Upon intravenous injection, this PET tracer will distribute in the body, where it can bind to a specifically chosen target. Eventually, radioactive decay of the tracer upon binding to the target leads to the emission of gamma rays, which can be detected and quantified (19). As PET itself does not provide anatomical information, this technique is often combined with computed tomography (CT) (20). At-present, 11 studies investigated PET-CT in patients with SSc-ILD (see **Table 1**).

**Table 1:** Summary of clinical positron emission tomography studies in patients with systemic sclerosis associated interstitial lung disease.

Study and patient groups	Radiotracer & target	Study design and main findings
Jacquelin et al., 2016 **(** 21 **)** ◼ **SSc-ILD (n = 1)** ◼ **Other CTD-ILD: IM (n = 2); iNSIP (n = 3); aSS (n = 1); pSS (n = 7); RA (n = 2); aSS + pSS (n = 1); and UCTD (n = 1)** Median (range) disease duration of 20 months (1–298)	^18^F-FDG GLUT1-4 Glucose metabolism	*Retrospective study* - All patients with a NSIP pattern on HRCT showed increased pulmonary uptake of ^18^F-FDG, the median extent was 19%. - ^18^F-FDG was observed in all HRCT identified lesions: consolidations (90%); ground-glass opacities (89%); honeycombing (85%) and reticulations (76%). - Pulmonary ^18^F-FDG uptake correlated with pulmonary function improvement under anti-inflammatory treatment, while HRCT fibrosis score and max uptake values did not.
Uehara et al., 2016 **(** 22 **)** ◼ **SSc-ILD (n = 9)** ◼ **Other CTD-ILD: DM/PM (n = 16); SLE (n = 2); RA (n = 7); MCTD (n = 4); SS (n = 2); AAV (n = 4); and PMR (n = 1)** Disease duration unknown	^18^F-FDG GLUT1-4 Glucose metabolism	*Retrospective study* - Visual score and pulmonary ^18^F-FDG uptake were higher in the active phase (n=32) compared to the inactive phase (n=37), independent of findings on CT and CTD subtype. Patients were considered at an active phase when immunosuppressive treatment was initiated or intensified. - Seventeen patients that were followed because of active phase showed a decrease in visual score and pulmonary ^18^F-FDG uptake after initiation or intensification of treatment. The two patients who died during follow-up showed higher visual uptake compared to the patients that were still alive. - Changes in visual score correlated with Krebs von Lungen-6 and CRP, while pulmonary ^18^F-FDG uptake did not.
Bellando et al., 2019 **(** 23 **)** ◼ **SSc (n = 7 of which 3 had ILD)** Median disease duration of 5 years	^18^F-FDG GLUT1-4 Glucose metabolism	*Retrospective study* - Pulmonary uptake of ^18^F-FDG was higher in patients with higher extent of ILD, measured both in HRCT positive and HRCT negative lesions. - Also, ^18^F-FDG uptake was higher in HRCT positive lesions than in HRCT negative lesions in patients with ILD.
Peelen et al., 2020 **(** 24 **)** ◼ **SSc-ILD (n = 5)** ◼ **Controls: SSc (n = 3), SLE (n = 8) and pSS (n = 4) without ILD** Mean disease duration of 5 years	^18^F-FDG GLUT1-4 Glucose metabolism	*Retrospective study* - Pulmonary uptake of ^18^F-FDG was higher in dorsobasal regions and as expressed in basal-apical ratios of patients with SSc-ILD when compared to patients without ILD. - Elevated pulmonary uptake of ^18^F-FDG coincided with both reticulation and ground-glass lesions on HRCT.
Ledoult et al., 2021 **(** 25 **)** ◼ **SSc (n = 36 of which 22 had ILD)** ◼ **Controls: Hodgkin Lymphoma (n = 89)** Median (range) disease duration of ILD was 2.0 years (0.0 - 8.5)	^18^F-FDG GLUT1-4 Glucose metabolism	*Retrospective study* - Pulmonary uptake of ^18^F-FDG was higher in SSc (in general) and SSc-ILD than in controls. No differences were found in regions outside of the lung (skin, lymph nodes, joints, muscles, and esophagus). - SSc patients with progressive ILD showed higher pulmonary uptake of ^18^F-FDG than patients with stable disease. - Pulmonary ^18^F-FDG uptake correlated with extent of fibrosis and pulmonary function tests.
Branley et al., 2008 **(** 26 **)** ◼ **SSc with fibrosing alveolitis (n = 15)** ◼ **Controls (n = 7)** Mean disease duration of 8 years	^11^C-[R]-PK11195 PBR Macrophages	*Prospective study* - A trend of reduced pulmonary uptake of ^11^C-[R]-PK11195 was seen in patients with SSc with fibrosing alveolitis when compared to controls. - Pulmonary uptake of ^11^C-[R]-PK11195 was inversely correlated to lung density, which was higher in patients with SSc with fibrosing alveolitis than in controls.
Adams et al., 2019 **(** 27 **)** ◼ **SSc-ILD (n = 1)** ◼ **Other CTD-ILD: RA (n = 3); aSS (n = 3); cEAA (n = 2); CTD (n = 1)** ◼ **Controls: RA without ILD (n = 5)** Disease duration of 3-6 years	[^89^Zr]Zr-rituximab CD20+ B cells	*Prospective study* - Pulmonary uptake of [^89^Zr]Zr-rituximab was visual and quantifiable in 4 patients with CTD-ILD after the administration of rituximab. The other patients with CTD-ILD did not show increased pulmonary uptake of this tracer. - In one patient with SSc-ILD uptake of [^89^Zr]Zr-rituximab was mainly seen in lymph nodes, patchy areas in the lung parenchyma and around hilar regions.
Adams et al., 2020 **(** 28 **)** ◼ **SSc-ILD (n = 2)** ◼ **Other CTD-ILD: cEAA (n = 7); RA (n = 4); aSS (n = 6); CTD (n= 1); DM (n=1)** Disease duration of 0-24 years	[^89^Zr]Zr-rituximab CD20+ B cells	*Prospective study* - Non-responders to Rituximab treatment showed higher splenic uptake of [^89^Zr]Zr-rituximab than responders. - Non-response was defined as a worsening clinical state, while responders improved or stabilized.
Lukey et al., 2020 **(** 29 **)** ◼ **SSc-ILD (n = 1)** ◼ **Other ILD: RA (n = 1); IPF (n = 7)** ◼ **Controls (n = 6)** Disease duration unknown	^18^F-FB-A20FMDV2 Integrin αvβ6	*Prospective study* - Uptake of ^18^F-FB-A20FMDV2 was mainly seen in fibrotic areas, and was higher in patients with ILD than in healthy controls. - ^18^F-FB-A20FMDV2 PET/CT repeated after 2 weeks showed reproducible results. - Specifically for SSc-ILD, uptake of the tracer was at lower end of the range, which was probably explained by the fact that only mild fibrotic changes were seen in the lungs of this patient.
Bergmann et al., 2021 **(** 30 **)** ◼ **SSc-ILD (n = 21)** ◼ **Controls (n = 21)** Mean disease duration of 5.5 years	^68^Ga-FAPI-04 FAP-α Activated fibroblasts	*Prospective* study - Pulmonary uptake of ^68^Ga-FAPI-04 was higher in SSc-ILD than in controls. - In SSc-ILD, pulmonary uptake of ^68^Ga-FAPI-04 was primarily seen in fibrotic lesions and was higher in patients with more extensive ILD and previous ILD progression. Baseline pulmonary uptake of ^68^Ga-FAPI-04 was independently associated with progression of ILD. - In 5 patients with follow-up, changes in pulmonary ^68^Ga-FAPI-04 uptake related to nintedanib response.
Röhrich et al., 2022 **(** 31 **)** ◼ **SSc-ILD (n = 1)** ◼ **Other ILD: IPF (n = 6); RA (n = 2); cPFE (n = 1); DIP (n = 1); IPAF (n = 2); uILD (n = 1); Sarcoidosis (n = 1)** All patients were suspected of lung cancer Disease duration unknown	^68^Ga-FAPI-46 FAP-α Activated fibroblasts	*Retrospective study* - Patients with ILD showed elevated uptake and high target to background values of ^68^Ga-FAPI-46 at 1 hour after injection in both fibrotic and cancer lesions. - Time activity curves differed between fibrotic and cancer lesions. - After correction for density, uptake of ^68^Ga-FAPI-46 correlated positively with fibrosis index and negatively with ground-glass opacity index.

AAV, ANCA associated vasculitis; aSS, anti-Synthethase Syndrome; cEAA, chronic Extrinsic Allergic Alveolitis; cPFE, combined Pulmonary Fibrosis and Emphysema; CT, Computed Tomography; CTD, Connective Tissue Disease; DIP, Desquamative Interstitial Pneumonia; DM/PM, Dermatomyositis/Polymyositis; FAP-α, Fibroblast Activation Protein Alpha; GLUT1-4, Glucose transporters 1-4; HRCT, High Resolution Computed Tomography; IM, Inflammatory Myositis; iNSIP, idiopathic Non-Specific Interstitial Pneumonia; ILD, interstitial lung disease; uILD, unclassifiable ILD; IPAF, Interstitial pneumonia with autoimmune features; IPF, Idiopathic pulmonary fibrosis; MCTD, Mixed Connective Tissue Disease; PBR, Pheripheral Benzodiazepine receptors; PET, Positron emission tomography; PMR, Polymyalgia Rheumatica; pSS, primary Sjogrens Syndrome; RA, Rheumatoid Arthritis; SLE, Systemic Lupus Erythematosus; SS, Sjogrens Syndrome; SSc, Systemic sclerosis; UCTD, Undifferentiated Connective Tissue Disease.

The most commonly used PET tracer is ^18^F-Fluorodeoxyglucose (FDG). FDG uptake is facilitated through glucose transporters, which therefore acts as a surrogate marker for glucose metabolism. Five studies retrospectively investigated ^18^F-FDG PET-CT in SSc-ILD (see **Table 1**) (21–25). In these studies, pulmonary uptake of ^18^F-FDG was higher in patients with SSc-ILD than in controls and correlated negatively with lung function tests. Also, ^18^F-FDG was detected in areas without established fibrosis on HRCT, suggesting subclinical activity. In addition, this tracer may reflect disease activity as higher uptake values were found in patients requiring treatment initiation or intensification compared to those who did not. Finally, ^18^F-FDG was found to be a predictor of ILD progression, and was sensitive to change under anti-inflammatory therapy (21, 22, 25). These results are in line with ^18^F-FDG PET-CT studies in other forms of ILD and stress the potential value of this modality to improve risk assessment and prediction of treatment response in patients with SSc-ILD (32–34).

## ^18^F-FDG PET-CT in the Context of Autologous Stem Cell Transplantation in Systemic Sclerosis

In order to investigate whether upfront aHSCT is associated with a higher event-free survival compared to conventional immunosuppressive treatment with intravenous cyclophosphamide pulses followed by oral mycophenolate mofetil in patients with early severe dcSSc, the UPSIDE study (UPfront autologous hematopoietic Stem cell transplantation versus Immunosuppressive medication in early DiffusE cutaneous systemic sclerosis; NCT04464434) was recently initiated (35). The patients in this study have a maximum disease duration of two years, starting from presentation of the first non-Raynaud phenomenon. Also, patients should have dcSSc with a high modified Rodnan Skin Score (≥15) and/or clinically significant pulmonary, cardiac or renal involvement. Within the UPSIDE study, the value of several biomarkers for prognosis and response to therapy are being investigated in sub studies, including biomarkers related to the presence of ILD.

One of these sub studies will investigate ^18^F-FDG PET-CT in patients with early severe dcSSc and ILD. In this sub study we aim to 1) compare pulmonary uptake of ^18^F-FDG between SSc-ILD and SSc without ILD 2) compare pulmonary uptake of ^18^F-FDG to HRCT lesions and regular clinical assessments 3) identify if pulmonary uptake of ^18^F-FDG is associated with treatment response and/or disease progression. ^18^F-FDG PET-CT will be performed at baseline and 12 months after randomization to either aHSCT or conventional immunosuppressive treatment. Fifteen patients with ILD and five patients without ILD (controls) will be included.

**Figure 1:**
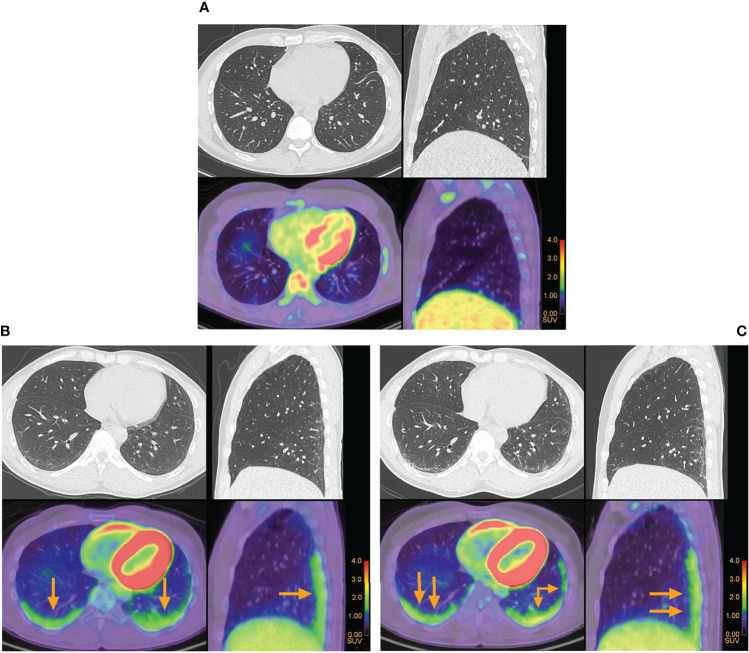
Pulmonary ^18^F-FDG positron emission tomography and high resolution computed tomography in patients with early severe diffuse cutaneous systemic sclerosis. **(A)** No visual uptake of ^18^F-FDG was observed in a patient with systemic sclerosis without interstitial lung disease. **(B)** Increased uptake of ^18^F-FDG in the lungs of a patient with systemic sclerosis associated interstitial lung disease is indicated by the arrows. **(C)** Repeated ^18^F-FDG positron emission tomography in the same patient, who had severe progression of interstitial lung disease after 3 months of cyclophosphamide pulse treatment. Higher uptake of ^18^F-FDG was seen at the follow-up scan when compared to baseline.

To illustrate the potential value of ^18^F-FDG PET-CT in this specific group of patients, we show images of ^18^F-FDG PET-CT and HRCT in two patients diagnosed with early severe dcSSc (**Figure 1**). **Figure 1A** shows no visual uptake of ^18^F-FDG in the lungs of a patient without ILD. **Figures 1B, C** show repeated ^18^F-FDG PET-CT of a patient with ILD. In this patient ^18^F-FDG PET-CT was performed to rule out malignancy and was repeated after 4 months because of severe progression of ILD despite 3 months of cyclophosphamide pulse treatment, before switching to aHSCT. In this patient, ^18^F-FDG uptake increased in dorsobasal lung areas, in this short period of time, illustrating that increase in ^18^F-FDG uptake is in line with clinical deterioration.

## Discussion

In this perspective, we have discussed and illustrated the potential value of ^18^F-FDG PET-CT in patients with SSc-ILD, specifically those with severe dcSSc and ILD in the context of aHSCT. The ^18^F-FDG PET-CT study that we are currently performing is important because we take into account several critical limitations of previous studies. First, this study will focus specifically on patients with SSc-ILD and will include a sufficient number of patients. Previous studies have not always focused specifically on patients with SSc-ILD, which makes it hard to draw conclusions for this specific group patients. Furthermore, patient numbers were often smaller than in our current study. Second, the disease duration of the included patients is short (maximum 2 years), which is important, because progressive ILD is mostly seen in the first five years after diagnosis (1). Finally, since this ^18^F-FDG PET-CT is performed within the frame of the UPSIDE study, we will have 5 years of proper follow-up next to the ^18^F-FDG PET-CT that is repeated after one year of treatment. As the ^18^F-FDG PET signal is quantifiable, this technique is suitable to demonstrate changes over time and (non)response to therapy. Not much is known about the specificity of ^18^F-FDG PET abnormalities for ILD, as comparative studies between ILD and other lung diseases, such as infection or malignancy, are currently lacking. However, this technique is typically paired with a (HR)CT, which can detect other lung diseases such as pneumonia and malignancy with high specificity, and are taken into account when evaluating ^18^F-FDG PET.

It is worth mentioning that several new PET tracers have been investigated in patients with SSc-ILD, including ^11^C-[R]-PK11195 (macrophages), [^89^Zr]Zr-rituximab (B-cells), ^18^F-FB-A20FMDV2 (integrins) and ^68^Ga-FAPI-04/46 (activated fibroblasts) (see **Table 1**) (26–31). Of these new tracers, ^68^Ga-FAPI-04 seems most promising as it was independently associated with ILD progression in a heterogeneous population of patients with SSc-ILD, and tracer uptake correlated to response to anti-fibrotic therapy with nintedanib (30). In pre-clinical studies, tracers that target apoptosis, vascular leakage, and fibronectin have been evaluated with varying results (36–38). PET has also been extensively evaluated in idiopathic pulmonary fibrosis, which is a progressive fibrotic form of ILD by definition (PF-ILD) (39), including the use of ^68^Ga-CBP8 (collagen type 1), ^68^Ga-pentixafor (C-X-C chemokine receptor 4), ^68^Ga-DOTA-NOC/TATE (somatostatin receptors) and ^18^F-FMISO (hypoxia) (40–44). Especially ^68^Ga-pentixafor showed promising results, as early changes (baseline-6 weeks) in pulmonary uptake of ^68^Ga-pentixafor after the initiation of pirfenidone correlated with forced vital capacity after 12 months, with higher uptake of ^68^Ga-pentixafor indicating a larger decline in lung function. Pirfenidone is a recently approved anti-fibrotic therapy for PF-ILD, that is currently under investigation for SSc-ILD as well (45). At this point, there are no comparative studies between ^18^F-FDG and any of these other tracers in SSc-ILD, which limits any conclusions on the role any of these tracers could play in the prediction of ILD progression and predicting treatment response in relation to ^18^F-FDG. As these tracers are more specific to pathophysiological processes in the lung, it would be advocated to use these tracers in future studies to unravel the mechanisms of action of aHSCT in the lungs of patients with severe dcSSc and ILD.

In summary, ^18^F-FDG PET-CT is a promising tool for the assessment of SSc-ILD, but needs further investigation. We will be the first to investigate ^18^F-FDG PET-CT in patients with early severe dcSSc and ILD in the context of aHSCT. The results of this study have to be awaited before drawing conclusions about the definite value of ^18^F-FDG PET-CT in this specific group of patients. With this study, we also hope to enrich our knowledge for the design of future studies with other tracers. For future research, it would be interesting to compare ^18^F-FDG to other, more specific tracers to see if they are superior, to investigate the mechanisms of action of antifibrotic and anti-inflammatory therapy, including aHSCT, in the lungs and whether ^18^F-FDG and these specific tracers can contribute to personalized treatment of patients with SSc-ILD.

## Data Availability Statement

The original contributions presented in the study are included in the article/supplementary material. Further inquiries can be directed to the corresponding author.

## Ethics Statement

The studies involving human participants were reviewed and approved by the VU University Medical Center, Amsterdam. The patients/participants provided their written informed consent to participate in this study.

## Author Contributions

BB, CL, and AV: conceptual design, drafting of the article, critical scientific revision, and approval of final version. GZ and BB: design of figures. GZ, EN, LM, JS, JV-B, and JL: critical revision and approval of the final version. All authors contributed to the article and approved the submitted version.

## Funding

Boehringer Ingelheim and the Dutch Arthritis Society (under grant number 21-1-201) (partially) funded the ^18^F-FDG PET-CT study within the UPSIDE study. The funder was not involved in the study design, collection, analysis, interpretation of data, the writing of this article or the decision to submit it for publication.

## Conflict of Interest

The authors declare that the research was conducted in the absence of any commercial or financial relationships that could be construed as a potential conflict of interest.

## Publisher’s Note

All claims expressed in this article are solely those of the authors and do not necessarily represent those of their affiliated organizations, or those of the publisher, the editors and the reviewers. Any product that may be evaluated in this article, or claim that may be made by its manufacturer, is not guaranteed or endorsed by the publisher.
